# Editorial Notes

**Published:** 1894-06

**Authors:** 


### Editorial Notes.

DR. W. L. STERLING, one of the oldest and most highly respected physicians of this city, died on June 1st.

---

DR. O. STOVALL, of this city, died May 29th. Dr. Stovall was over eighty years old, and had spent the greater part of his life in active practice in Atlanta.

---

DRS. J. McF. GASTON and Willis F. Westmoreland were in attendance at the meeting of the Congress of American Physicians and Surgeons, Washington, D. C., last week.

---

DR. R. O. COTTER, of Barnesville, paid us a call last week. Dr. Cotter is now in New York, and will sail for Europe in a few weeks to be absent for a year. The Doctor will favor the RECORD with a letter each month.

---

THE Masonic Hospital Association, of Chicago, has removed to the Champlain building, corner State and Madison streets. Dr. G. Frank Lydston is Medical Director and Surgeon in charge, and reports the hospital to be in a most flourishing condition.

---

DR. EMORY LANPHEAR, for many years editor of the *Kansas City Medical Index*, has resigned the Chair of Operative Surgery and Clinical Surgery in the Kansas City Medical College, and has removed to St. Louis. He makes the change in order to become Professor of Surgery in the St. Louis College of Physicians and Surgeons, one of the oldest and strongest medical schools of the West.

